# Knowledge, Awareness, and Perceptions Regarding Autism Among Parents in Karachi, Pakistan

**DOI:** 10.7759/cureus.3299

**Published:** 2018-09-13

**Authors:** Muhammad Salar Anwar, Mahnoor Tahir, Khushboo Nusrat, Muhammad R Khan

**Affiliations:** 1 Department of Internal Medicine, Dow University of Health Sciences (DUHS), Karachi, PAK

**Keywords:** autism spectrum disorders, autism, autism awareness, autism knowledge, parenting, autistic

## Abstract

Background

The prevalence of autism is growing worldwide. Owing to parents being the primary caregivers in most situations, their ability to recognize the signs and symptoms of autism and respond appropriately is of paramount importance in aiming to provide the best healthcare to autistic individuals. This study was conducted with the aim of ascertaining the parent’s knowledge and awareness of autism.

Methods

A cross-sectional survey was conducted among parents residing in Karachi, Pakistan. We excluded any individuals belonging to the medical profession, those who have autistic children, and those who couldn’t completely comprehend English and Urdu. A sample size of 339 parents was selected. A validated and pre-tested questionnaire was administered among the study participants to record demographic information, knowledge, and perceptions regarding autism and its signs and symptoms. Data were analyzed using Statistical Package for Social Sciences (SPSS version 23.0, IBM Corp., Armonk, NY, US). A knowledge score was calculated for opinions about autism and its sign and symptoms individually to reflect a participant’s overall knowledge regarding autism.

Results

From our study population, 75% of our population had heard of autism, with those who knew of someone with the disorder displaying greater awareness. However, our participants displayed poor knowledge scores, with a mean score of 5.59 in the section concerning correct opinions on autism and that of 6.84 in the section testing knowledge of signs and symptoms. Despite this, 95.6% of the participants were willing to get their children treated, in the event of them being diagnosed with autism.

Conclusion

Unfortunately, our population displayed a lack of awareness and knowledge regarding autism. To fill this gap, awareness programs should be conducted to promote parent’s knowledge regarding autism, so as to allow for early diagnoses and an appropriate treatment plan/therapy. On a positive note, most were willing to get their children tested and treated in case of a diagnosis. However, only a small number of participants knew of autism centers in Karachi. General practitioners are needed to play a key role in counseling parents about autism.

## Introduction

Autism spectrum disorder (ASD) is a neurodevelopmental disorder that can be diagnosed around the age of two [1]. It consists of autism disorder, Asperger’s syndrome, and pervasive developmental disorder. This article will focus on autism disorder only. Autism is more common in males and children born prematurely and it has a strong link to the genetic disorder, fragile X [1-2]. Of the childhood population in Mexico, 0.87% have been diagnosed with this disease whereas 1% have been diagnosed in South Thames UK [3-4]. Its prevalence is increasing over the last few years and one in 45 children in the US is born with it [5]. The reason for the increasing numbers could be due to the inclusion/diagnostic criteria modifying over time to be more inclusive and vast or because of increased risk factors being associated with the disorder itself. The alarming signs include delayed language development, repetitive behavior, non-responsiveness to their names, and communication delays. Parents of diagnosed autistic children complain of disturbed sleep pattern, resulting in overall drastic effects of daytime dysfunction, all this contributing to very high stress levels in parents/caregivers [6-7].

There needs to be adequate awareness of autism disorder. The reason for individuals to be well informed is because family members of autistic children undergo great financial and mental burden and the more uninformed they are, the greater the risk of misdiagnoses, thus making their child more difficult and resistant to therapy. Earlier recognition and diagnosis will help parents in devising a well-constructed and streamlined treatment plan, helping release stress, as they will be able to discuss and share their burden with the appropriate doctor and research a correct diagnosis. There is a high chance of misdiagnosis or late diagnosis if there is a lack of awareness about the signs of this disorder, especially among parents since they will be the first to observe any unusual behavior compared to other children or siblings of the same age group. An early and accurate diagnosis plays a massive role in outcomes and improvement of behavior in the child. If the parent recognizes the symptoms of autism in their child, like lack of eye contact, hyperactivity, increased attachments to toys, no reaction to verbal cues, etc., they can seek medical help, and according to the Diagnostic and Statistical Manual of Mental Disorders, Fifth Edition (DSM 5), have accurate knowledge regarding the status of their child. After a parent’s child is diagnosed correctly, they will be able to focus on their child’s betterment, and as the demands and teaching strategy of autistic children is unique, they can approach the child’s school teachers with ease about the disorder and work together towards the child’s education simultaneously with therapy, resulting in an improvement in their diagnosed child’s communication skills, etc.

We aimed to assess the knowledge of the signs and symptoms of this disease in parents who don't have autistic children. The aim of this study was to find out the gap in awareness and the lack of information among the general population in Pakistan, especially since the estimated number of autistic children in this country is one out of 120 [8]. This resulted in knowing if awareness and knowledge are up to the mark, thus helping in decreasing the burden of parents and teachers and eradicating any confusion and discomfort regarding the child’s behavior.

## Materials and methods

A cross-sectional, questionnaire-based study was conducted among the urban dwellers of Karachi, Pakistan, from 20 January 2018 to 31 March 2018 to evaluate autism-related knowledge, signs, symptoms, and the treatment regime. A sample size of 250 was calculated using Open Epi, however, 350 people were included in this study to get a better representation of knowledge among the population. In total, 11 participants failed to complete the survey and so those forms were discarded, giving a response rate of 96.86%.

Eligible study participants included urban dwellers of Karachi living in Gulshan-e-Iqbal between the age of 18-70 years, who had children. We excluded any individual belonging to the medical profession, those who have autistic children, and those who couldn’t completely comprehend English and Urdu.

A 34-item questionnaire was designed following an extensive literature review. Furthermore, the questionnaire was also translated into Urdu, the native language. The questionnaire was adapted from similar studies [9]. A pilot study was conducted on a convenient sample of 40 people to assess the clarity and comprehension of the questionnaire. The ambiguous questions were omitted and the questionnaire was refined based on the results of the pilot study.

The questionnaire consisted of four sections. The first section included sociodemographic details of each participant, which included age, gender, qualification, profession, and number of children. The second section consisted of 12 questions, which assessed participants' knowledge and perception regarding autism, for example, whether it’s an inherited disorder, a mental disorder, preventable, a life-long condition, and other behavioral habits of an autistic child. The signs and symptoms of autism were evaluated in the third section and included specific questions, such as delayed response to name, fails to show interest in other children, emotional reciprocity, inappropriate attachment to toys, limited attention span, etc. All questions were based on the multiple-choice format and included options of “agree,” “disagree,” and “I don’t know.” The final section determined knowledge related to treatment regimens and participants were further asked if they would be willing to do a diagnostic test for autism on their children, and if they were diagnosed, would they be willing to seek the appropriate treatment measures.

The data was entered and interpreted using Statistical Package for Social Sciences (SPSS version 23.0, IBM Corp; Armonk, NY, US). Individual responses were counted and displayed using percentages and frequency. A knowledge score was calculated to reflect a participant’s opinions about autism and its signs and symptoms by assigning one mark to each correct answer. These were then tabulated individually. We assigned a knowledge score of eight out of 12 (66.7%) and that of nine out of 13 (69.2%) as a good score, for opinions about autism and its signs and symptoms, respectively. Each of these knowledge scores was then analyzed with different sociodemographic factors, such as age, gender, profession, etc. Associations among these variables were tested individually using non-parametric independent sample tests. A P-value less than 0.05 was considered significant.

## Results

Our study consisted of a population of 339 parents living in Karachi. As presented in Table 1, out of the study population, 186 (54.9%) were females while the rest were males. The mean age of the respondents was calculated at 50.6±10.3. Almost all of the participants (89.1%, n=309) were married. Respondents ranged from different educational qualifications with graduates (67.0%, n=226) being the most common. Among the socioeconomic classes, participants were mainly from the middle-class background (n= 155, 68.6%) followed by the upper (17.1%, n=58) and then lower classes (13.3%, n=45). When asked about profession, housewife (30.1%, n=102) was the most common response followed by businessman and teacher with a percentage of 15.0% (n=51) and 13.3% (n=45), respectively. One hundred eight (n=31.9%) of the parents had two children while only 8% (n=27) of the parents had five children or more. Females scored a statistically significant higher score for both opinions and signs and symptoms of autism with a p-value of 0.048 and 0.032, respectively. Unmarried parents also scored significantly higher for both knowledge scores than the married population (p=0.000, p=0.001). Among the category of number of children, parents having five or more children scored significantly the highest among them (p=0.000) in the knowledge score of opinions on autism only. Statistically significant associations were found in the sociodemographics of profession (p=0.014), academic qualifications (p=0.016), and socioeconomic status (p=0.000) with engineers/unemployed, graduates, and upper-class population scoring the highest in their respective category.

**Table 1:** Sociodemographics of the Study Population

Socio-demographic characteristics	Percentages n (%)	Mean of correct responses about opinions on autism	P-value (<0.05)	Average correct identification of signs and symptoms	P-value (<0.05)
Gender			.048		.032
Male	153 (45.1%)	5.16 ± 3.35		6.25 ± 4.39	
Female	186 (54.9%)	5.95 ± 2.84		7.32 ± 3.76	
Marital Status			.000		.001
Married	302 (89.1%)	5.36 ± 3.15		6.57 ± 4.16	
Unmarried	37 (10.9%)	7.51 ± 1.74		9.08 ± 2.52	
Socioeconomic			.258		.000
Upper	58 (17.1%)	6.19 ± 3.02		8.60 ± 4.38	
Middle	236 (69.6%)	5.23 ± 3.03		6.59 ± 3.82	
Lower	45 (13.3%)	5.16 ± 3.47		5.87 ± 4.47	
Qualifications			.070		.016
Graduate	227 (67.0%)	5.79 ± 2.87		7.07 ± 3.81	
Undergraduate	86 (25.4%)	5.62 ± 3.26		6.99 ± 4.29	
Secondary School	26 (7.7%)	3.81 ± 3.98		4.31 ± 4.95	
Profession			.125		.014
Housewife	102 (30.1%)	6.41 ± 2.67		7.41 ± 3.38	
Businessman	51 (15.0%)	5.18 ± 3.57		6.24 ± 4.55	
Engineer	30 (8.8%)	5.80 ± 2.68		8.00 ± 3.69	
Unemployed	21 (6.2%)	4.86 ± 3.73		8.00 ± 5.37	
Teacher	45 (13.3%)	5.13 ± 2.66		7.13 ± 4.01	
Management	27 (8.0%)	4.78 ± 3.81		4.78 ± 4.31	
Others	63 (18.6%)	5.43 ± 3.10		6.14 ± 4.07	
No. of Children			.000		.087
One	48 (14.2%)	5.38 ± 3.07		6.75 ± 4.35	
Two	108 (31.9%)	6.31 ± 3.06		7.56 ± 4.14	
Three	90 (26.5%)	4.53 ± 3.28		5.97 ± 4.53	
Four	66 (19.5%)	5.59 ± 2.75		6.55 ± 3.43	
Five or more	27 (8.0%)	6.67 ± 2.54		7.78 ± 2.62	

Table 2 shows that among the parents participating in our study, three-quarters of them had previously heard about autism. There was a significant difference in the mean of correct opinions and an identification of signs with regard to autism among the two groups (p=0.000), with people who had heard previously about autism scoring higher than their counterpart. Media was the most popular source of knowledge among them, with 33.9% of the parents gaining awareness from there. Parents who had gained their knowledge from doctors and health professionals had a significantly higher mean for correct opinions and signs and symptoms 7.47 (p=0.000) and 8.74 (p=0.000), respectively. Parents who had prior knowledge of someone undergoing autism treatment had a higher mean of correct responses regarding both opinions and symptomology (p=0.000).

**Table 2:** Prior Awareness of Autism Among Parents

Previous Knowledge	Frequency (n, %)	Mean of correct responses about opinions on autism	P-value (<0.05)	Average correct identification of signs and symptoms	P-value (<0.05)
Heard About Autism			.000		.000
Yes	255 (75.2%)	6.73 ± 2.17		8.19 ± 3.19	
No	84 (24.8%)	2.14 ± 2.94		2.75 ± 3.79	
Source of Knowledge			.000		.000
Media	115 (33.9%)	6.42 ± 2.21		8.23 ± 3.41	
Books\Magazines	47 (13.9%)	7.19 ± 2.55		8.70 ± 2.78	
Relatives	52 (15.3%)	6.42 ± 2.15		7.54 ± 3.23	
School\ University	22 (6.5%)	7.05 ± 1.62		7.91 ± 2.88	
Doctors\ Health Professionals	19 (15.6%)	7.47 ± 1.71		8.74 ± 3.02	
Knowledge of anyone undergoing autism treatment			.000		.000
Yes	57 (16.8%)	7.32 ± 1.73		9.37 ± 2.56	
No	282 (83.2%)	5.24 ± 3.20		6.34 ± 4.15	

**Figure 1:**
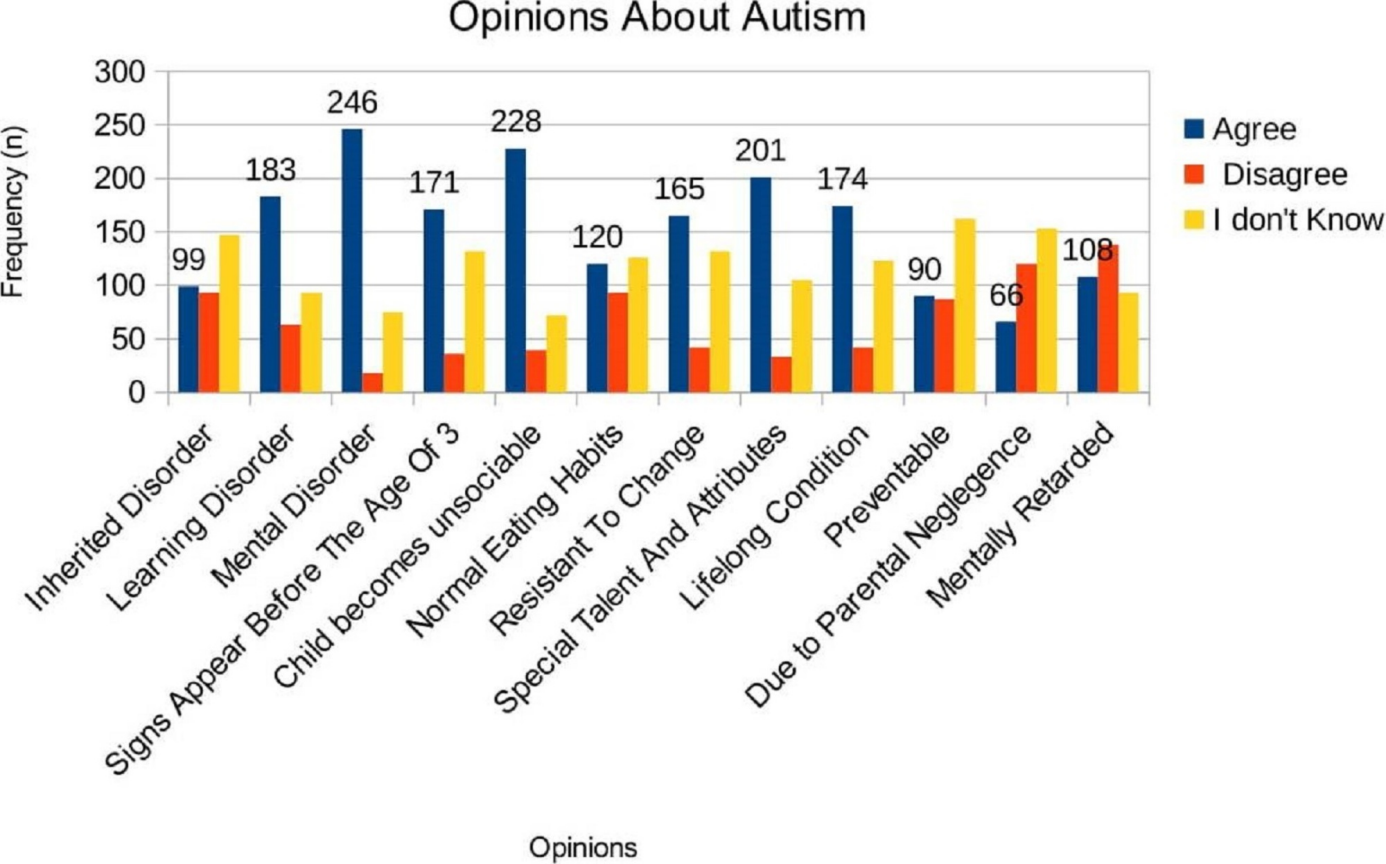
Opinions of Parents about Autism

Figure 1 illustrates the responses of parents regarding different opinions on autism. Parents mostly agreed upon autism being a mental disorder (n=246, 72.6%), the child becomes unsociable (n=228, 67.3%), and an autistic child has special talents/attributes (n=201, 59.3%). A significant population of parents (n=108, 31.9%) disagreed on considering an autistic child mentally retarded. Upon different opinions on autism, the parents were unsure and responded with “I don’t know,” especially in the cases of considering autism an inherited disorder (n=147, 43.4%), if autism is preventable (n=162, 47.8%), and if autism is caused due to parental negligence (n=153, 45.1%).

In Table 3, the knowledge of different signs and symptoms of autism can be assessed. Of the parents, 63.7% (n=216) agreed that an autistic child is obsessed with the same routine and becomes upset at minor changes; 61.1% (n=207) agreed upon delayed language development and lack of interest in interacting with other children to be a sign of autism; and 61.9% (n=210) correctly disagreed that an autistic child makes good eye contact and uses appropriate hand gestures while interacting with other kids. An autistic child having no perception of fear or danger was the least sign that parents were aware of (37.2%, n=126). About 79.6% (n=270) people consider parental counseling to be an effective treatment for autism while 47.8 % (n=162) of the population considered diet to play no part in the treatment of autism. Only 39 (11.5%) knew that an autistic child can be tested clinically. Two hundred seven (61.1%) parents were willing to get their child tested for autism while 95.6% (n=324) of the population admitted that they were willing to get their child treated in case of them being diagnosed with autism. Only a few (15.9%, n=54) people were aware of autism centers present in Karachi.

**Table 3:** Knowledge of Different Signs and Symptoms of Autism Among Parents

Serial No.	Signs and Symptoms	Agree	Disagree	I Don’t Know
1.	An autistic child looks at other children when interacting with them or makes good eye contact.	36 (10.6%)	210 (61.9%)	93 (27.4%)
2.	Makes good and appropriate use of hand and body gestures when having conversations.	30 (8.8%)	210 (61.9%)	99 (29.2%)
3.	Fails to show interest in other children or has no interest in interacting with other children	207 (61.1%)	54 (15.9%)	78 (23.0%)
4.	Has emotional reciprocity (awareness about others being happy, sad, angry, etc. and responds appropriately).	87 (25.7%)	123 (36.3%)	129 (38.1%)
5.	Language development is delayed.	207 (61.1%)	21 (6.2%)	111 (32.7%)
6.	Has repetitive behavior.	198 (58.4%)	18 (5.3%)	123 (36.3%)
7.	Delayed response to name.	156 (46.0%)	45 (13.3%)	138 (40.7%)
8.	Does not respond to emotional cues, i.e., to affection.	159 (46.9%)	57 (16.8%)	123 (36.3%)
9.	Inappropriate attachment to certain toys or objects (prefers to play with the same toy for hours).	183 (54.0%)	12 (3.5%)	144 (42.5%)
10.	Has no perception of fear or danger.	126 (37.2%)	66 (19.5%)	147 (43.4%)
11.	Gets upset at even minor changes in routine, obsessed with the same routine.	216 (63.7%)	0 (0.0%)	123 (36.3%)
12.	Uses repetitive phrases at odd or inappropriate times, like singing an advertisement jingle.	144 (42.5%)	30 (8.8%)	165 (48.7%)
13.	The attention span of an autistic child is deficient or limited	186 (54.9%)	30 (8.8%)	123 (36.3%)

## Discussion

Despite significant effort, there is insufficient data concerning the prevalence of autism owing to factors such as the way autism is perceived and an inability to diagnose autism [10]. Several studies stress on the early diagnosis and thus early intervention in patients with autism spectrum disorder (ASD), which may lead to better outcomes in some patients [11-12]. As signs and symptoms become apparent after children reach 18 months, our study aimed to assess the level of awareness regarding autism among parents.

To give our assessment greater accuracy, we calculated knowledge scores for the sections concerning correct opinions and signs and symptoms. Though our participants weren’t completely unaware of autism, they displayed poor knowledge in both sections. A mean score of 5.59 was seen for the section regarding correct opinions for autism, with eight out of 13 being considered a good score, and a mean score of 6.84 for signs and symptoms was calculated, with a good score being decided as nine out of 14. The poor knowledge displayed by our participants is similarly seen in other studies, such as one conducted in Pakistan among pre-school teachers, of which only 50% were able to identify a majority of disease characteristics and one where only 17% of pre-school teachers being interviewed in China could answer more than 50% of the items accurately [13-14]. However, studies showing better awareness have also been conducted, such as a study among first-grade nursing and medical students in Istanbul where 70.9% were moderately aware of autism [15].

According to our study, a greater number of females identified correct opinions regarding autism in comparison to males, enforcing the same finding seen in studies conducted in Karachi [16]. Better responses were also noted regarding correct opinion about autism by unmarried participants and those with five children or more. In analyzing the ability to identify the signs and symptoms of autism, females and unmarried participants again possessed greater knowledge, in line with previous findings [16]. Pickard and Ingersoll found that parents of children with autism and of a high socioeconomic status were more aware of service options for their child in comparison to parents of a lower socioeconomic status, which supports our finding of parents from the upper class identifying more signs and symptoms of autism [17]. Our study showed variable results in relation to the impact profession has on the ability to identify signs and symptoms with the unemployed and engineers displaying the most knowledge and those employed in management displaying the least, as similarly noted in a Jordan-based study, with the level of education having no effect on their behavior modification skills towards their autistic child [18].

Approximately 75% of our participants had heard about autism. These individuals could more effectively recognize correct opinions and signs and symptoms in relation to those participants who had never heard of autism. These findings are reinforced by a study conducted in Kerala, which assessed the level of awareness concerning autism among parents before and after an awareness program was held, with a greater level of knowledge seen among parents after the program [19]. Not surprisingly, media proved to be the greatest source of knowledge owing to its popularity [20], but healthcare professionals proved to be a more useful source. As detailed by Hansen, our study also proved that participants who knew someone undergoing treatment for autism were more knowledgeable [21].

According to the American Psychiatry Association, autism is a neurodevelopmental disorder diagnosed in childhood, which leads to impaired social skills and difficulty in adapting to change and is a disorder that can be managed but not cured [22]. Among our participants, the majority managed to identify these characteristics but incorrectly agreed with autistic patients possessing normal eating habits, as existing literature suggests problems in feeding due to compulsive behaviors, motor or sensory difficulties, and gastrointestinal problems [23]. As the concept of autistic patients possessing savant or special abilities, such as excellence in mathematics, art, music, and rote learning, is frequently portrayed in the media and in books, it isn’t surprising that the majority of our population agreed with this point. Researchers have looked into this particular characteristic repeatedly and though every person with ASD isn’t said to possess these skills, Howlin et al. concluded that one-third of autistic patients do so [24]. Even though parental negligence has been established as having a relation to autism [22], our population remained unsure on this point, along with the disease being inherited and preventable, which matches the degree of uncertainty in the literature available [25]. Even though most diagnosed cases of autism are idiopathic, secondary causes, such as German measles, have been identified as well.

Well-documented and established signs and symptoms of autism reported on by multiple sources [22,26] are an inability to interact with other children, a delay in or no development of important milestones, such as speaking and responding to names, obsessive repetitive behavior, difficulty in adapting to change and reacting to emotions, all of which were recognized correctly by a majority of our participants. Autistic children don’t make eye contact or use gestures [26], which were features our population was aware of. Despite being known symptoms of autism, participants were not aware of patients having a diminished perception of danger and response to emotions. This results from their inability to decipher what another person might be feeling, as they don’t interpret changes in expression and tone [25]. The management of ASD is mainly focused on psychological interventions, and the benefits of providing this treatment as soon as possible have been stressed on repeatedly. As parents are the primary caregivers in most situations, training parents on methods of treatment is known to have a benefit and, over the years, detailed research is being done on this to develop specific training techniques [27]. Most of the parents we interviewed felt parental counseling is an effective method of treatment but didn’t feel that diet had any effect on the condition despite evidence conflicting with this opinion, with particular importance being placed on gluten- and casein-free diets [28]. Unfortunately, a minority claimed autism could be diagnosed clinically, despite specific tests, such as the childhood autism rating scale (CARS) and autism behavior checklist (ABC), being the only method of diagnosis [25]. However, on a positive note, parents agreed to get their child tested and, if needed, treated for autism but were unaware of centers in Karachi, a finding seen repeatedly among studies such as one conducted in China among teachers [13].

Certain limitations were met within our study owing to convenience sampling. A majority of our participants were from the middle class, resulting in an inefficiency in assessing the effect socioeconomic status has on the level of awareness among the parents. Other than that, more females participated in our study in comparison to males. As autism is still a relatively undiscussed disorder among the Pakistani population, many people were completely unaware of the disorder and were not willing to respond to any questions regarding it. As this study was carried out in Karachi only, a clear estimation of the awareness regarding autism of the entire Pakistani population cannot be made. However, our study is the first of its kind in Pakistan, as it is conducted on parents and the results we obtained can be used to evaluate how to bridge the existing gap in knowledge among parents, thus allowing them to know which signs to look out for and how to seek appropriate guidance.

## Conclusions

It can safely be concluded that there is a lack of awareness and insufficient knowledge about autism among parents. Other than the signs and symptoms, parents are also unaware of diagnosis and treatment methods. This results in delayed identification and intervention, leading to unsatisfactory outcomes in patients. As parents are the primary caregivers, significant efforts should be aimed at raising knowledge levels amongst them regarding autism, through awareness campaigns. Health professionals should also be directed to educate new parents on all details concerning autism.
